# Supplementary material for: Learning curve and surgical outcome of robotic assisted colorectal surgery with ERAS program
Source: Sci Rep. 2022 Nov 29;12:20566. doi: 10.1038/s41598-022-24665-w (PMC9709162; doi:10.1038/s41598-022-24665-w)
Supplement: Supplementary file 2 — Supplementary Information 2. [file 41598_2022_24665_MOESM2_ESM.zip › RAL Raw data-Ñ[▒K/052/HS_ñw╝╨░O▒Kñσ.pdf]

正式摘要(電子簽章：000000208565722)

W102 / W102 ☐ 英文標題(注意:無英文姓名-請補病人基本檔!) ☐【調取】憑證

臺中榮民總醫院

出院病歷摘要

轉入號： 轉出號：

[redacted]

(8)流水編號	
(9)入院日期	110/01/15 (2021)
(10)轉科日期	
(11)出院日期	110/01/21 (2021) 住院天數計 6 日

診斷

- 入院 :rectal cancer
- 出院 :rectal cancer s/p robot assisted low anterior resetion and loop ileostomy on 2021/1/16
  Urinary tract infection

主訴

- difficulty of defecation with blood blot for 1 month

病史

- This 31 years old female patient didn't have special past history. She complained difficulty of defecation with blood blot for about 1 month. She came to our hospital for help and rectal tumor was found and malignancy was proved in biopsy. There was no elevated tumor marker. Abdominal CT showed tumor sized about 3.2cm and invades perirectal tissues. She also came to GYN OPD for fertility consult. Under the impression of rectal adenocarcinoma, she was admitted for surgical treatment.

體檢發現

- PS 70%,
  - General appearance :
    - easy looking , conscious clear , without cardiopulmonary distress
  - HEENT :
    - Eyes: no anemic conjunctiva, no icteric sclera, normal EOM
    - ENT: no otorrhea, no rhinorrhea, no erythematous mucosa, no injected tonsil, no deformity
  - Neck: supple, freely movable, trachea midline,no deformity, no engorged jugular vein , no palpable thyroid or lymph node enlargement
  - Chest:
    - Inspection: Grossly normal, symmetric and fully expansion, no accessory muscle used, no operation scar, no spider angiomata, no gynecomastia
    - Palpation: no palpable mass or nodule, no subcutaneous emphysema, no tenderness
    - Ausculation: clear breathing sound
  - Heart:
    - Percussion: normal heart size
    - Ausculation: regular heart beat, normal S1, S2, no gallop, no murmur
  - Abdomen:
    - Inspection: in
      - flat contour, symetrically, no scar, no stria, no dilated veins, no rashes
    - Ausculation: normoactive bowel sound, no abdominal bruits
    - palpation: soft, no muscle guarding or rigidity, no local tenderness, no rebounding pain, no palpable mass, no palpable pulsation
    - Percussion: no shifting dulllness, no hepatomegaly, no splenomagaly
  -

Peripheral pulsation :	CA	BA	RA	FA	PA	PTA	DPA
Right side :	++	++	++	++	++	++	++/+++
Left side :	++	++	++	++	++	++	++/+++

  - Genito-anus: no tenderness or palpable mass lesion or rectal shelf, normal external genitalia, normal anal tone, brown stool

CRS CONDITION

  -
    - Lung

手術日期及方法

- 手術日期及方法20210116 Robot Assisted Low Anterior Resection + loop ileostomy

住院治療經過

- After admission, operation with robot assisted low anterior resetion and loop ileostomy was done on 2021/1/16 as scheduled. We let her try diet as ERAS protocol. Ostomy rod was removed on 1/20. Under the stable condition, she was discharged on 1/21.

合併症

- nil

一般檢查

- 累積報告 :

DATE	TIME	ALB	TP	BIL.T	BIL.D	ALKP	AST	ALT	LDH	CHOL	TG	HDL-C	LDL	UA	NH3	AMY
1100116	1659	3.5		0.56	0.22	75	31	42								

DATE	TIME	WBC	RBC	HGB	HCT	MCV	PLT	BLST	NEUT	BAND	SEG	LYM	MON	EOS	BAS
1100116	1659	24060	3.99	12.6	36.5	91.5	341				96.0	1.0	2.0	0.0	0.0
1100121	0907	8060	3.50	11.4	32.4	92.6	320				76.8	13.8	5.7	3.2	0.5

DATE	TIME	NA	K	CL	CA	BUN	CREAT	Mg	P	CRP	ESR	PCT	LIPASE	LACTATE
1100116	1659	139	4.1	108	8.1	8	0.68							

DATE	TIME	GLUCOSE	PAB	TRF	ZN	CU	KETONE	CK	CKMB	PT-P	PT-C	APTT-P	APTT-C	TRO-T
1100116	1659	138												

DATE	TIME	SP-GR	PH	PROT	GLUC	KETON	BILI	UROB	OB	NITR	LEU	RBC	WBC	EPCEL
1100119	0057	1.006	6.5	Negative	Negative	Negative	Negative	0.2	1+	Positive	2+	0-2	35-50	6-10

- 檢驗項目 :**Bacterial Culture(Ordinary) 檢體:Urine-voided midstream 報告日期 :1100121**
  附註 :
  陰性結果 :
  陽性結果 : ISOLATED ORGANISM: COLONY COUNT:( )/ml
  (1) Escherichia coli >100000
  (2) Gram negative bacillus 2000
  (3)
  菌落量定義 : P:Pure H:Heavy M:Moderate S:Slight moderate F:Few E:Enrich
  SUSCEPTIBILITY TEST: S:Susceptible I:Intermediate R:Resistant D: Susceptible Dose Dependent (SDD) NS: Nonsusceptible

	(1)	(2)	(3)	(4)
Ampicillin				
Cefazolin	R>=64			
Clindamycin				
Clarithromycin				
Gentamicin	S=1			
Amikacin	S=2			
Chloramphenicol				

- Ampicillin/Sulbactam R>=32
  Penicillin
  Gentamicin(120ug)
  Trimethoprim+Sulfamethoxazole R>=320
  Vancomycin
  Oxacillin
  Cefpirome
  Ciprofloxacin S=0.25
  Minocycline
  Carbapen (IPM) S=0.25
  Cefoperazone/Sulbactam S=8
  Cefotaxime
  Ceftazidime I=8
  Cefepime S=0.12
  Ceftriaxone R=32
  Flomoxef S=8
  Levofloxacin
  Teicoplanin
  Piperacillin/Tazobactam S=4
  Cefmetazole
  Aztreonam
  Tigecycline
  Amoxicillin/Clavulanic acid
  Cefoxitin R=32
  Metronidazole
  Carbapen (MPM)
  Erythromycin
  Moxifloxacin
  Linezolid
  Ertapenem S=0.12
  Colistin
  Daptomycin
  REMARK
  注意事項： For Enterobacteriaceae :

1.對於腸內菌屬的敏感性試驗，cefepime若採用CLSI M100-24的判讀標準，當試驗結果為敏感性時，其建議治療劑量為1gm Q12H；若以最低抑制濃度（MIC）的方法，結果為敏感劑量依賴性（susceptible-dose dependent：SDD）時，當MIC為 4 ug/mL時則建議劑量為1gm/Q8H或2gm/Q12H，若是MIC為8 ug/mL時或採用紙錠擴散法結果為SDD，則建議治療劑量為2gm/Q8H。

2.Cefazolin predict results for the oral cephalosporins agents (cefaclor, cephalexin) when used for therapy of uncomplicated UTIs due to E. coli, K. pneumoniae, and P. mirabilis.

3.Cefoperazone/Sulbactam目前並無國際性的判讀標準，現行引用文獻的判讀規範，僅供臨床參考。

檢驗項目 **:Chemical Exam.** 檢體**:Urine** 報告日期 **:1100119**
CHEM: SP.GR. 1.006 (1.003-1.035)
PH 6.5 (5.0-8.0)
PROTEIN Negative (NEGATIVE) (+/-: 15- 22.49 mg/dl;
+: 22.5-64.9 mg/dl; 2+: 65-199.9 mg/dl; 3+: ≧200 mg/dl)
GLUCOSE Negative mg/dL (NEGATIVE)
KETONE Negative (NEGATIVE)
BILIRUBIN Negative (NEGATIVE)
O.B. 1+ (NEGATIVE)
NITRITE Positive (NEGATIVE)
UROBILI 0.2 (0.1-1.0)
LEU 2+ (NEGATIVE)
COLOR Yellow (Yellow)
CLEARITY Cloudy (CLEAR)
備註:
Remark :

檢驗項目 **:Microscopic Exam.** 檢體**:Urine** 報告日期 **:1100119**
MICROSCOPIC EXAM.:
RBC. 0-2 /HPF (0-2)
WBC&/OR PUS CELL. 35-50 /HPF (M:0-2, F:0-5)
EPITH.CELL. 6-10 /HPF (Variable)
CAST. /LPF (Negative~a few Hyaline cast)
/LPF
CRYSTAL. /HPF (Variable)
/HPF
/HPF
/HPF
Others BACTERIA +
%
備註:
Remark :

特殊檢查

病理報告

- 活體組織切片病理報告 手術日期：**109/12/23** 病理號：**S0942066**

診斷醫師:王仁卿 報告日期:109/12/28
Pathologic diagnosis:
Rectum, endoscopic biopsy -- Adenocarcinoma, moderately differentiated.
Gross description:
The specimen consists of multiple pieces of tan soft tissue, up to 0.2x0.2x0.2 cm. All for section.
Microscopic description:
Section shows rectal mucosa with invasive carcinoma in abortive glandular structure and desmoplastic reaction.
Note:
1. The case has been peer reviewed by two doctors.
2. The diagnosis is concurred in intradepartment consensus meeting on 2020/12/25.
#T-59600_2 #M-81403_2 0 1340 00CM0F

其它

出院時情況

- 3.改門診治療

出院指示

藥名	劑量	劑量單位	用法	頻次	數量	數量單位	現況	天數	單位
Ultracet tab	1	TAB	PO	Q6H	28	TAB	TH	7	天
U-Clor cap 250mg	2	CAP	PO	Q8H	42	CAP	TH	7	天
UroGEN tab 50mg	1	TAB	PO	TIDAC	21	TAB	TH	7	天

出院計劃

主治醫師_2062F 蔣鋒帆_____ / 住院醫師_2331F 陳凱祥_____

正式摘要(電子簽章：**000000208565722)**

W102 / W102

doctorRank:B
